# Checklist of the infraorder Tipulomorpha (Trichoceridae, Tipuloidea) (Diptera) of Finland

**DOI:** 10.3897/zookeys.441.7533

**Published:** 2014-09-19

**Authors:** Jukka Salmela, Andrius Petrašiūnas

**Affiliations:** 1 Metsähallitus, Natural Heritage Services, P.O. Box 8016, FI-96101 Rovaniemi, Finland; 2 Vilnius university Faculty of Natural Sciences Department of Zoology Ciurlionio st. 21/27 LT 03101 Vilnius, Lithuania

**Keywords:** Checklist, Finland, Diptera, Trichoceridae, Pediciidae, Limoniidae, Cylindrotomidae, Tipulidae

## Abstract

A checklist of the infraorder Tipulomorpha: families Trichoceridae, Pediciidae, Limoniidae, Cylindrotomidae and Tipulidae (Diptera) recorded from Finland.

## Introduction

Trichoceridae is a small family of medium sized gnats, mostly of Holarctic distribution. Larvae live in different decaying matter like leaves, wood, fungi or dead mammals. Adults form numerous swarms that are encountered during colder fall and spring months or warmer winter days in warmer climates or during the short summer months in northern latitudes.

No comprehensive and up to date key exists for north European adults or larvae, but a combination of Shtackelberg (1988), Starý (1999) and Petrašiūnas (2009) could be used. However, Shtackelberg (1988) must be used with caution because several species have since been published. List of trichocerids presented here is based on literature review (see Dahl 1968, Dahl and Alexander 1976), no new material has been studied.

Tipuloidea crane flies (Pediciidae, Limoniidae, Cylindrotomidae and Tipulidae) belong to the infraorder Tipulomorpha together with trichocerids (Wiegmann et al. 2011). Following Starý (1992), most European authors distinguish four families: Limoniidae, Tipulidae, Pediciidae and Cylindrotomidae. North American authors, strongly influenced by C.P. Alexander, usually prefer a single family, Tipulidae; other families, *sensu* Starý, are there subfamilies or tribes (Byers 1992, Petersen et al. 2010). According to a recent phylogenetic analysis (Petersen et al. 2010), two families are recognized, Tipulidae and Pediciidae, but the consensus tree of that analysis had many unresolved polytomies. Crane fly larvae occur usually in moist or (semi)aquatic habitats, but larvae may also be found on mosses, in dry soil, fungal fruiting bodies and dead wood. Cylindrotomids are herbivorous, feeding on vascular plants or mosses. Most larvae of (semi)aquatic species have open spiracles and are dependent on aerial oxygen supply, but truly aquatic species are able to withdraw dissolved oxygen through the cuticle of tracheal gills.

An annotated list of Finnish crane flies was provided by Salmela (2012a) and a few species were later added in subsequent papers (Salmela 2012b, 2013). Species richness of Finnish crane flies is negatively correlated with latitude but mire-dwelling crane flies have anomal species richness gradient (Salmela 2012). Finnish crane flies are rather well known, but perhaps up to 20 more species could be found from Finland (Salmela 2013).

**Table 1.** Number of species by family.

Family	Number of species in			Level of knowledge
	World (Oosterbroek 2014)	Europe (Pape et al. 2011)	Finland	
**Tipulomorpha:**				
Trichoceridae	183	50	15–17	poor-average
Pediciidae	490	63	19	good
Limoniidae	10528	545	196	good
Cylindrotomidae	71	7	7	good
Tipulidae	4280	456	114–115	good

## Checklist

Nematocera Dumeril, 1805

infraorder Tipulomorpha Rohdendorf, 1961

**TRICHOCERIDAE** Róndani, 1841

***CLADONEURA*** Scudder, 1894

= ***Diazosma*** Bergroth, 1913

? *Cladoneura hirtipenne* (Siebke, 1863)

***TRICHOCERA*** Meigen, 1803

**sg. *Metatrichocera*** Dahl, 1966

*Trichocera gigantea* (Dahl, 1967)

*Trichocera lutea* Becher, 1886

*Trichocera mackenziei* (Dahl, 1967)

**sg. *Saltrichocera*** Krzeminska, 2002

*Trichocera annulata* Meigen, 1818

? *Trichocera arctica* Lundström, 1915

*Trichocera implicata* Dahl, 1976

*Trichocera maculipennis* Meigen, 1818

*Trichocera parva* Meigen, 1804

*Trichocera regelationis* (Linnaeus, 1758)

*Trichocera rufescens* Edwards, 1921

*Trichocera saltator* (Harris, 1776)

= *fuscata* Meigen, 1818

*Trichocera sparsa* Starý & Martinovský, 1996

**sg. *Trichocera*** Meigen, 1803

*Trichocera hiemalis* (De Geer, 1776)

*Trichocera inexplorata* (Dahl, 1967)

*Trichocera major* Edwards, 1921

*Trichocera sibirica* Edwards, 1920

**PEDICIIDAE** Osten Sacken, 1860

PEDICIINAE Osten Sacken, 1860

***DICRANOTA*** Zetterstedt, 1838

**sg. *Dicranota*** Zetterstedt, 1838

*Dicranota bimaculata* (Schummel, 1829)

*Dicranota crassicauda* Tjeder, 1972

*Dicranota guerini* Zetterstedt, 1838

**sg. *Paradicranota*** Alexander, 1934

*Dicranota gracilipes* Wahlgren, 1905

*Dicranota pavida* (Haliday, 1833)

*Dicranota robusta* Lundström, 1912

*Dicranota subtilis* Loew, 1871

**sg. *Rhaphidolabis*** Osten Sacken, 1869

*Dicranota exclusa* (Walker, 1848)

***PEDICIA*** Latreille, 1809

**sg. *Crunobia*** Kolenati, 1859

*Pedicia straminea* (Meigen, 1838)

**sg. *Pedicia*** Latreille, 1809

*Pedicia rivosa* (Linnaeus, 1758)

***TRICYPHONA*** Zetterstedt, 1837

**sg. *Tricyphona*** Zetterstedt, 1837

*Tricyphona immaculata* (Meigen, 1804)

*Tricyphona livida* Madarassy, 1881

*Tricyphona schummeli* Edwards, 1921

*Tricyphona unicolor* (Schummel, 1829)

ULINAE Alexander, 1920

***ULA*** Haliday, 1833

**sg. *Ula*** Haliday, 1833

*Ula bolitophila* Loew, 1869

*Ula kiushiuensis* Loew, 1869

*Ula mixta* Starý, 1983

*Ula mollissima* Haliday, 1833

= *inconclusa* (Walker, 1856)

*Ula sylvatica* (Meigen, 1818)

**LIMONIIDAE** Speiser, 1909

CHIONEINAE Róndani, 1841

***ARCTOCONOPA*** Alexander, 1955

*Arctoconopa forcipata* (Lundström, 1915)

*Arctoconopa obscuripes* (Zetterstedt, 1851)

*Arctoconopa zonata* (Zetterstedt, 1851)

***CHEILOTRICHIA*** Rossi, 1848

**sg. *Cheilotrichia*** Rossi, 1848

*Cheilotrichia imbuta* (Meigen, 1818)

**sg. *Empeda*** Osten Sacken, 1869

*Cheilotrichia areolata* (Lundström, 1912)

*Cheilotrichia cinerascens* (Meigen, 1804)

= *nubila* (Schummel, 1829)

*Cheilotrichia neglecta* (Lackschewitz, 1927)

***CHIONEA*** Dalman, 1816

**sg. *Chionea*** Dalman, 1816

*Chionea araneoides* Dalman, 1816

= *minuta* Tahvonen, 1932

*Chionea crassipes* Boheman, 1846

**sg. *Sphaeconophilus*** Becker, 1912

*Chionea lutescens* Lundström, 1907

= *brevirostris* Tahvonen, 1932

***CRYPTERIA*** Bergroth, 1913

*Crypteria limnophiloides* Bergroth, 1913

***ERIOCONOPA*** Stary, 1976

*Erioconopa diuturna* (Walker, 1848)

*Erioconopa trivialis* (Meigen, 1818)

***ERIOPTERA*** Meigen, 1803

**sg. *Erioptera*** Meigen, 1803

*Erioptera beckeri* Kuntze, 1914

= *fuscipennis* misid.

*Erioptera divisa* (Walker, 1848)

*Erioptera flavata* (Westhoff, 1882)

= *gemina* Tjeder, 1967

*Erioptera griseipennis* Meigen, 1838

*Erioptera lutea* Meigen, 1804

*Erioptera nielseni* Meijere, 1921

*Erioptera pederi* Tjeder, 1969

*Erioptera sordida* Zetterstedt, 1838

*Erioptera squalida* Loew, 1871

*Erioptera tordi* Tjeder, 1973

***GNOPHOMYIA*** Osten Sacken, 1860

*Gnophomyia acheron* Alexander, 1950

*Gnophomyia lugubris* (Zetterstedt, 1838)

*Gnophomyia viridipennis* (Gimmerthal, 1847)

***Gonempeda*** Alexander, 1924

*Gonempeda flava* (Schummel, 1829)

***GONOMYIA*** Meigen, 1818

**sg. *Gonomyia*** Meigen, 1818

*Gonomyia abscondita* Lackschewitz, 1935

= *lucidula* misid.

*Gonomyia bifida* Tonnoir, 1920

*Gonomyia dentata* Meijere, 1920

*Gonomyia simplex* Tonnoir, 1920

*Gonomyia stackelbergi* Lackschewitz, 1935

*Gonomyia tenella* (Meigen, 1818)

**sg. *Teuchogonomyia*** Alexander, 1968

*Gonomyia edwardsi* Lackschewitz, 1925

***HOPLOLABIS*** Osten Sacken, 1869

**sg. *Parilisia*** Savchenko, 1976

*Hoplolabis areolata* (Siebke, 1872)

*Hoplolabis vicina* (Tonnoir, 1920)

***MOLOPHILUS*** Curtis, 1833

**sg. *Molophilus*** Curtis, 1833

*Molophilus appendiculatus* (Staeger, 1840)

*Molophilus ater* (Meigen, 1804)

*Molophilus bifidus* Goetghebuer, 1920

*Molophilus bihamatus* Meijere, 1918

*Molophilus cinereifrons* Meijere, 1920

*Molophilus corniger* Meijere, 1920

*Molophilus crassipygus* Meijere, 1918

= *ochrescens* Edwards, 1938

*Molophilus flavus* Goetghebuer, 1920

*Molophilus griseus* (Meigen, 1804)

*Molophilus medius* Meijere, 1918

*Molophilus obscurus* (Meigen, 1818)

*Molophilus occultus* Meijere, 1918

*Molophilus ochraceus* (Meigen, 1818)

*Molophilus propinquus* (Egger, 1863)

*Molophilus pullus* Lackschewitz, 1927

***NEOLIMNOPHILA*** Alexander, 1920

*Neolimnophila carteri* (Tonnoir, 1921)

*Neolimnophila placida* (Meigen, 1830)

***ORMOSIA*** Rondani, 1856

**sg. *Oreophila*** Lackschewitz, 1935

*Ormosia sootryeni* (Lackschewitz, 1935)

**sg. *Ormosia*** Rondani, 1856

*Ormosia brevinervis* (Lundström, 1907)

*Ormosia clavata* (Tonnoir, 1920)

*Ormosia depilata* Edwards, 1938

*Ormosia fascipennis* (Zetterstedt, 1838)

*Ormosia hederae* (Curtis, 1835)

*Ormosia lineata* (Meigen, 1804)

*Ormosia loxia* Starý, 1983

*Ormosia pseudosimilis* (Lundström, 1912)

*Ormosia ruficauda* (Zetterstedt, 1838)

*Ormosia staegeriana* Alexander, 1953

***PHYLLOLABIS*** Osten Sacken, 1877

*Phyllolabis macroura* (Siebke, 1863)

***RHABDOMASTIX*** Skuse, 1890

*Rhabdomastix borealis* Alexander, 1924

*Rhabdomastix laeta* (Loew, 1873)

*Rhabdomastix parva* (Siebke, 1863)

***RHYPHOLOPHUS*** Kolenati, 1860

*Rhypholophus haemorrhoidalis* (Zetterstedt, 1838)

*Rhypholophus varius* (Meigen, 1818)

***SCLEROPROCTA*** Edwards, 1938

*Scleroprocta pentagonalis* (Loew, 1973)

*Scleroprocta sororcula* (Zetterstedt, 1851)

= *danica* (Nielsen, 1923)

***SYMPLECTA*** Meigen, 1830

**sg. *Psiloconopa*** Zetterstedt, 1838

*Symplecta lindrothi* (Tjeder, 1955)

*Symplecta meigeni* (Zetterstedt, 1838)

*Symplecta stictica* (Meigen, 1818)

**sg. *Symplecta*** Meigen, 1830

*Symplecta chosenensis* (Alexander, 1940)

*Symplecta hybrida* (Meigen, 1804)

*Symplecta mabelana* (Alexander, 1955)

*Symplecta scotica* (Edwards, 1938)

**sg. *Trimicra*** Osten Sacken, 1861

*Symplecta pilipes* (Fabricius, 1787)

***TASIOCERA*** Skuse, 1890

**sg. *Dasymolophilus*** Goetghebuer, 1920

*Tasiocera exigua* Savchenko, 1973

*Tasiocera fuscescens* (Lackschewitz, 1940)

*Tasiocera murina* (Meigen, 1818)

LIMNOPHILINAE Bigot, 1854

***ADELPHOMYIA*** Bergroth, 1891

*Adelphomyia punctum* (Meigen, 1818)

***AUSTROLIMNOPHILA*** Alexander, 1920

**sg. *Archilimnophila*** Alexander, 1934

*Austrolimnophila harperi* (Alexander, 1926)

*Austrolimnophila unica* (Osten Sacken, 1869)

***DICRANOPHRAGMA*** Osten Sacken, 1860

**sg. *Brachylimnophila*** Alexander, 1966

*Dicranophragma adjunctum* (Walker, 1848)

*Dicranophragma separatum* (Walker, 1848)

= *nemorale* misid.

= *leucophaea* misid.

***ELOEOPHILA*** Rondani, 1856

*Eloeophila maculata* (Meigen, 1804)

*Eloeophila mundata* (Loew, 1971)

*Eloeophila submarmorata* (Verrall, 1887)

*Eloeophila trimaculata* (Zetterstedt, 1838)

*Eloeophila verralli* (Bergroth, 1912)

***EPIPHRAGMA*** Osten Sacken, 1860

**sg. *Epiphragma*** Osten Sacken, 1860

*Epiphragma ocellare* (Linnaeus, 1761)

***EUPHYLIDOREA*** Alexander, 1972

*Euphylidorea dispar* (Meigen, 1818)

= *lineola* misid.

*Euphylidorea meigeni* (Verrall, 1886)

*Euphylidorea phaeostigma* (Schummel, 1829)

***EUTONIA*** van der Wulp, 1874

*Eutonia barbipes* (Meigen, 1804)

***HEXATOMA*** Latreille, 1809

**sg. *Hexatoma*** Latreille, 1809

*Hexatoma fuscipennis* (Curtis, 1836)

= *nubeculosa* misid.

***IDIOPTERA*** Macquart, 1834

*Idioptera linnei* Oosterbroek, 1992

= *fasciata* (Linnaeus, 1767) preocc.

*Idioptera pulchella* (Meigen, 1830)

= pulchella var. macropteryx (Tjeder, 1955)

***LIMNOPHILA*** Macquart, 1834

**sg. *Limnophila*** Macquart, 1834

*Limnophila pictipennis* (Meigen, 1818)

*Limnophila schranki* Oosterbroek, 1992

= *punctata* (Schrank, 1781)

***NEOLIMNOMYIA*** Seguy, 1937

**sg. *Neolimnomyia*** Seguy, 1937

*Neolimnomyia batava* (Edwards, 1938)

***PARADELPHOMYIA*** Alexander, 1936

**sg. *Oxyrhiza*** de Meijere, 1946

*Paradelphomyia fuscula* (Loew, 1873)

*Paradelphomyia nigrina* (Lackschewitz, 1940)

***PHYLIDOREA*** Bigot, 1854

**sg. *Macrolabina*** Savchenko, 1986

*Phylidorea nigronotata* (Siebke, 1870)

**sg. *Paraphylidorea*** Savchenko, 1986

*Phylidorea fulvonervosa* (Schummel, 1829)

= *Euphylidorea lineola* misid.

**sg. *Phylidorea*** Bigot, 1854

*Phylidorea abdominalis* (Staeger, 1840)

*Phylidorea bicolor* (Meigen, 1804)

*Phylidorea ferruginea* (Meigen, 1818)

*Phylidorea heterogyna* (Bergroth, 1913)

*Phylidorea longicornis* (Schummel, 1829)

= *glabricula* (Meigen, 1830)

*Phylidorea nervosa* (Schummel, 1829)

= *nigricollis* (Meigen, 1830)

*Phylidorea squalens* (Zetterstedt, 1838)

*Phylidorea umbrarum* (Krogerus, 1937)

***PILARIA*** Sintenis, 1889

*Pilaria decolor* (Zetterstedt, 1851)

*Pilaria discicollis* (Meigen, 1818)

*Pilaria meridiana* (Staeger, 1840)

*Pilaria nigropunctata* (Agrell, 1945)

= *fuscipennis* misid.

*Pilaria scutellata* (Staeger, 1840)

***PSEUDOLIMNOPHILA*** Alexander, 1919

**sg. *Pseudolimnophila*** Alexander, 1919

*Pseudolimnophila lucorum* (Meigen, 1818)

LIMONIINAE Speiser, 1909

***ACHYROLIMONIA*** Alexander, 1965

*Achyrolimonia decemmaculata* (Loew, 1873)

***ANTOCHA*** Osten Sacken, 1860

**sg. *Antocha*** Osten Sacken, 1860

*Antocha vitripennis* (Meigen, 1830)

***ATYPOPHTHALMUS*** Brunetti, 1911

**sg. *Atypophthalmus*** Brunetti, 1911

*Atypophthalmus inustus* (Meigen, 1818)

***DICRANOMYIA*** Stephens, 1829

**sg. *Dicranomyia*** Stephens, 1829

*Dicranomyia aperta* Wahlgren, 1904

*Dicranomyia autumnalis* (Staeger, 1840)

*Dicranomyia consimilis* (Zetterstedt, 1838)

*Dicranomyia didyma* (Meigen, 1804)

*Dicranomyia distendens* Lundström, 1912

*Dicranomyia frontalis* (Staeger, 1840)

*Dicranomyia halterata* Osten Sacken, 1869

*Dicranomyia handlirschi* Lackschewitz, 1928

*Dicranomyia hyalinata* (Zetterstedt, 1851)

*Dicranomyia longipennis* (Schummel, 1829)

*Dicranomyia mitis* (Meigen, 1830)

*Dicranomyia modesta* (Meigen, 1818)

*Dicranomyia moniliformis* Doane, 1900

*Dicranomyia omissinervis* Meijere, 1918

*Dicranomyia patens* Lundström, 1907

*Dicranomyia radegasti* Starý, 1993

*Dicranomyia sera* (Walker, 1848)

*Dicranomyia terraenovae* Alexander, 1920

*Dicranomyia ventralis* (Schummel, 1829)

*Dicranomyia zernyi* Lackschewitz, 1928

**sg. *Glochina*** Meigen, 1830

*Dicranomyia liberta* Osten Sacken, 1860

*Dicranomyia tristis* (Schummel, 1829)

= *subtristis* Alexander, 1924

= *schineri* misid.

= *schineriana* misid.

**sg. *Idiopyga*** Savchenko, 1987

*Dicranomyia danica* Kuntze, 1919

*Dicranomyia esbeni* (Nielsen, 1940)

*Dicranomyia halterella* Edwards, 1921

*Dicranomyia intricata* Alexander, 1927

*Dicranomyia klefbecki* (Tjeder, 1941)

*Dicranomyia lulensis* (Tjeder, 1969)

*Dicranomyia magnicauda* Lundström, 1912

*Dicranomyia murina* (Zetterstedt, 1851)

*Dicranomyia ponojensis* Lundström, 1912

*Dicranomyia stigmatica* (Meigen, 1830)

**sg. *Melanolimonia*** Alexander, 1965

*Dicranomyia caledonica* Edwards, 1926

*Dicranomyia morio* (Fabricius, 1787)

*Dicranomyia occidua* Edwards, 1926

*Dicranomyia rufiventris* (Strobl, 1901)

*Dicranomyia stylifera* Lackschewitz, 1928

**sg. *Numantia*** Bigot, 1854

*Dicranomyia fusca* (Meigen, 1804)

***DICRANOPTYCHA*** Osten Sacken, 1860

*Dicranoptycha cinerascens* (Meigen, 1818)

*Dicranoptycha fuscescens* (Schummel, 1829)

***DISCOBOLA*** Osten Sacken, 1865

*Discobola annulata* (Linnaeus, 1758)

*Discobola caesarea* (Osten Sacken, 1854)

***ELEPHANTOMYIA*** Osten Sacken, 1860

**sg. *Elephantomyia*** Osten Sacken, 1860

*Elephantomyia edwardsi* Lackschewitz, 1932

*Elephantomyia krivosheinae* Savchenko, 1976

***HELIUS*** Lepeletier & Serville, 1828

**sg. *Helius*** Lepeletier & Serville, 1828

*Helius flavus* (Walker, 1856)

*Helius longirostris* (Meigen, 1818)

*Helius pallirostris* Edwards, 1921

***LIBNOTES*** Westwood, 1876

**sg. *Afrolimonia*** Alexander, 1965

*Libnotes ladogensis* (Lackschewitz, 1940)

***LIMONIA*** Meigen, 1803

**sg. *Limnobia*** Meigen, 18018

*Limonia badia* (Walker, 1848)

*Limonia flavipes* (Fabricius, 1787)

*Limonia macrostigma* (Schummel, 1829)

*Limonia maculicosta* (Coquillett, 1905)

*Limonia messaurea* Mendl, 1971

*Limonia nubeculosa* Meigen, 1804

*Limonia phragmitidis* (Schrank, 1781)

= *tripunctata* Fabricius, 1781

*Limonia stigma* (Meigen, 1818)

*Limonia sylvicola* (Schummel, 1829)

*Limonia trivittata* (Schummel, 1829)

***LIPSOTHRIX*** Loew, 1873

*Lipsothrix ecucullata* Edwards, 1938

*Lipsothrix errans* (Walker, 1848)

***METALIMNOBIA*** Matsumura, 1911

**sg. *Metalimnobia*** Matsumura, 1911

*Metalimnobia bifasciata* (Schrank, 1781)

*Metalimnobia charlesi* Salmela & Starý, 2008

*Metalimnobia quadrimaculata* (Linnaeus, 1761)

*Metalimnobia quadrinotata* (Meigen, 1818)

*Metalimnobia tenua* Savchenko, 1976

*Metalimnobia zetterstedti* (Tjeder, 1968)

= *elegans* Zetterstedt, 1838 preocc.

***NEOLIMONIA*** Alexander, 1964

*Neolimonia dumetorum* (Meigen, 1804)

***ORIMARGA*** Osten Sacken, 1869

**sg. *Orimarga*** Osten Sacken, 1869

*Orimarga attenuata* (Walker, 1848)

*Orimarga juvenilis* (Zetterstedt, 1851)

***RHIPIDIA*** Meigen, 1818

**sg. *Rhipidia*** Meigen, 1818

*Rhipidia maculata* Meigen, 1818

= *duplicata* (Doane, 1900)

*Rhipidia uniseriata* Schiner, 1864

**CYLINDROTOMIDAE** Schiner, 1863

***CYLINDROTOMA*** Macquart, 1834

*Cylindrotoma borealis* Peus, 1952

*Cylindrotoma distinctissima* (Meigen, 1818)

*Cylindrotoma nigriventris* Loew, 1849

***DIOGMA*** Edwards, 1938

*Diogma caudata* Takahashi, 1960

*Diogma glabrata* (Meigen, 1818)

***PHALACROCERA*** Schiner, 1863

*Phalacrocera replicata* (Linnaeus, 1758)

***TRIOGMA*** Schiner, 1863

*Triogma trisulcata* (Schummel, 1829)

**TIPULIDAE** Latreille, 1802

CTENOPHORINAE Osten Sacken, 1887

tribe Ctenophorini Osten Sacken, 1887

***CTENOPHORA*** Meigen, 1803

**sg. *Ctenophora*** Meigen, 1803

*Ctenophora flaveolata* (Fabricius, 1794)

*Ctenophora guttata* Meigen, 1818

*Ctenophora nigriceps* (Tjeder, 1949)

*Ctenophora pectinicornis* (Linnaeus, 1758)

***DICTENIDIA*** Brulle, 1833

*Dictenidia bimaculata* (Linnaeus, 1761)

***PHOROCTENIA*** Coquillett, 1910

*Phoroctenia vittata* (Meigen, 1830)

tribe Tanypterini Savchenko, 1966

***TANYPTERA*** Latreille, 1804

**sg. *Tanyptera*** Latreille, 1804

*Tanyptera atrata* (Linnaeus, 1758)

*Tanyptera nigricornis* (Meigen, 1818)

DOLICHOPEZINAE Osten Sacken, 1887

***DOLICHOPEZA*** Curtis, 1825

**sg. *Dolichopeza*** Curtis, 1825

*Dolichopeza albipes* (Strom, 1768)

*Dolichopeza bifida* Osterbrook & Lantsov, 2011

= *nitida* of authors

TIPULINAE Latreille, 1802

tribe Prionocerini Savchenko, 1966

***PRIONOCERA*** Loew, 1844

*Prionocera abscondita* Lackschewitz, 1933

*Prionocera chosenicola* Alexander, 1945

= *dimidiata* misid.

= *absentiva* misid.

*Prionocera pubescens* Loew, 1844

*Prionocera recta* Tjeder, 1948

= *lapponica* Tjeder, 1948

= *lackschewitzi* Mannheims, 1951

*Prionocera ringdahli* Tjeder, 1948

*Prionocera serricornis* (Zetterstedt, 1838)

*Prionocera subserricornis* (Zetterstedt, 1851)

= *proxima* Lackschewitz, 1933

*Prionocera turcica* (Fabricius, 1787)

*Prionocera woodoorum* Brodo, 1987

tribe Tipulini Latreille, 1802

***ANGAROTIPULA*** Savchenko, 1961

*Angarotipula tumidicornis* (Lundström, 1907)

***NEPHROTOMA*** Meigen, 1803

*Nephrotoma aculeata* (Loew, 1871)

*Nephrotoma analis* (Schummel, 1833)

*Nephrotoma appendiculata* (Pierre, 1919)

*Nephrotoma cornicina* (Linnaeus, 1758)

*Nephrotoma crocata* (Linnaeus, 1758)

*Nephrotoma dorsalis* (Fabricius, 1781)

*Nephrotoma flavescens* (Linnaeus, 1758)

*Nephrotoma lundbecki* (Nielsen, 1907)

*Nephrotoma lunulicornis* (Schummel, 1833)

*Nephrotoma pratensis* (Linnaeus, 1758)

*Nephrotoma quadristriata* (Schummel, 1833)

*Nephrotoma relicta* (Savchenko, 1973)

*Nephrotoma scurra* (Meigen, 1818)

*Nephrotoma submaculosa* Edwards, 1928

*Nephrotoma tenuipes* (Riedel, 1910)

***NIGROTIPULA*** Hudson & Vane-Wright, 1969

*Nigrotipula nigra* (Linnaeus, 1758)

***TIPULA*** Linnaeus, 1758

**sg. *Acutipula*** Alexander, 1924

*Tipula fulvipennis* De Geer, 1776

*Tipula maxima* Poda, 1761

**sg. *Arctotipula*** Alexander, 1934

*Tipula salicetorum* Siebke, 1870

= *nigricornis* Zetterstedt, 1851

**sg. *Beringotipula*** Savchenko, 1961

*Tipula unca* Wiedemann, 1817

= *hortensis* Meigen, 1818

**sg. *Dendrotipula*** Savchenko, 1964

*Tipula flavolineata* Meigen, 1804

**sg. *Emodotipula*** Alexander, 1966

*Tipula obscuriventris* Strobl, 1900

**sg. *Lindnerina*** Mannheims, 1965

*Tipula bistilata* Lundström, 1907

*Tipula subexcisa* Lundström, 1907

**sg. *Lunatipula*** Edwards, 1931

*Tipula affinis* Schummel, 1833

*Tipula circumdata* Siebke, 1863

= *livida* misid.

*Tipula fascipennis* Meigen, 1818

*Tipula humilis* Staeger, 1840

*Tipula laetabilis* Zetterstedt, 1838

= *dilatata* Schummel, 1833

*Tipula limitata* Schummel, 1833

*Tipula lunata* Linnaeus, 1758

= *luna* mistake

? *Tipula peliostigma* Schummel, 1833

*Tipula recticornis* Schummel, 1833

*Tipula selene* Meigen, 1830

*Tipula trispinosa* Lundström, 1907

*Tipula vernalis* Meigen, 1804

**sg. *Odonatisca*** Savchenko, 1956

*Tipula nodicornis* Meigen, 1818

= *juncea* Meigen, 1818

**sg. *Platytipula*** Matsumura, 1916

*Tipula luteipennis* Meigen, 1830

*Tipula melanoceros* Schummel, 1833

**sg. *Pterelachisus*** Róndani, 1842

*Tipula cinereocincta* Lundström, 1907

*Tipula crassicornis* Zetterstedt, 1838

*Tipula irrorata* Macquart, 1826

*Tipula jutlandica* Nielsen, 1947

*Tipula kaisilai* Mannheims, 1954

*Tipula laetibasis* Alexander, 1934

*Tipula luridorostris* Schummel, 1833

Tipula matsumuriana ssp. pseudohortensis Lackschewitz, 1932

*Tipula mutila* Wahlgren, 1905

*Tipula octomaculata* Savchenko, 1964

*Tipula pabulina* Meigen, 1818

*Tipula pauli* Mannheims, 1964

*Tipula pseudoirrorata* Goetghebuer, 1921

*Tipula recondita* Pilipenko & Salmela, 2012

*Tipula stenostyla* Savchenko, 1964

*Tipula submarmorata* Schummel, 1833

= *meigeni* Mannheims, 1966

*Tipula truncorum* Meigen, 1830

*Tipula varipennis* Meigen, 1818

= *pseudovariipennis* misid.

*Tipula wahlgreni* Lackschewitz, 1925

*Tipula winthemi* Lackschewitz, 1932

**sg. *Savtshenkia*** Alexander, 1965

*Tipula alpium* Bergroth, 1888

*Tipula benesignata* Mannheims, 1954

*Tipula confusa* van der Wulp, 1883

= *marmorata* Meigen, 1818

*Tipula gimmerthali* Lackschewitz, 1925

*Tipula grisescens* Zetterstedt, 1851

*Tipula interserta* Riedel, 1913

*Tipula invenusta* Riedel, 1919

*Tipula limbata* Zetterstedt, 1838

*Tipula obsoleta* Meigen, 1818

*Tipula pagana* Meigen, 1818

*Tipula signata* Staeger, 1840

*Tipula subnodicornis* Zetterstedt, 1838

**sg. *Schummelia*** Edwards, 1931

*Tipula variicornis* Schummel, 1833

**sg. *Tipula*** Linnaeus, 1758

*Tipula paludosa* Meigen, 1830

= *oleracea* misid.

*Tipula subcunctans* Alexander, 1921

= *czizeki* de Jong, 1925

**sg. *Vestiplex*** Bezzi, 1924

*Tipula excisa* Schummel, 1833

*Tipula hortorum* Linnaeus, 1758

*Tipula laccata* Lundström & Frey, 1916

*Tipula montana* Curtis, 1834

*Tipula nubeculosa* Meigen, 1804

*Tipula pallidicosta* Pierre, 1924

*Tipula scripta* Meigen, 1830

*Tipula sintenisi* Lackschewitz, 1933

*Tipula tchukchi* Alexander, 1934

= *bo* Mannheims, 1967

**sg. *Yamatotipula*** Matsumura, 1916

*Tipula chonsaniana* Alexander, 1945

*Tipula coerulescens* Lackschewitz, 1923

*Tipula couckei* Tonnoir, 1921

*Tipula fendleri* Mannheims, 1963

*Tipula freyana* Lackschewitz, 1936

*Tipula lateralis* Meigen, 1804

*Tipula marginella* Theowald, 1980

= *marginata* Meigen, 1818

*Tipula moesta* Riedel, 1919

*Tipula montium* Egger, 1863

*Tipula pierrei* Tonnoir, 1921

= *solstitialis* Westhoff, 1879

*Tipula pruinosa* Wiedemann, 1817

*Tipula quadrivittata* Staeger, 1840

## Excluded species

*Trichocera japonica* Matsumura, 1915

Twenty-two previously reported crane fly species were deleted from the Finnish list by Salmela (2012a); these are not repeated here.
